# B-Cell Receptor-Associated Protein 31 Negatively Regulates the Expression of Monoamine Oxidase A *Via* R1

**DOI:** 10.3389/fmolb.2020.00064

**Published:** 2020-04-30

**Authors:** Cong-cong Jia, Guoxun Li, Rui Jiang, Xia Liu, Qing Yuan, Weidong Le, Yue Hou, Bing Wang

**Affiliations:** ^1^College of Life and Health Sciences, Northeastern University, Shenyang, China; ^2^Center for Clinical Research on Neurological Diseases, The First Affiliated Hospital, Dalian Medical University, Dalian, China; ^3^Liaoning Provincial Key Laboratory for Research on the Pathogenic Mechanisms of Neurological Diseases, The First Affiliated Hospital, Dalian Medical University, Dalian, China; ^4^Key Laboratory of Data Analytics and Optimization for Smart Industry, Ministry of Education, Northeastern University, Shenyang, China

**Keywords:** Bap31, MAOA, iTRAQ, R1, X-linked syndrome

## Abstract

B-cell receptor-associated protein 31 (Bap31) is a three *trans*-membrane protein of the endoplasmic reticulum (ER). Patients who have loss of function of Bap31 suffered from X-linked syndrome, such as motor and intellectual disabilities, dystonia, and sensorineural deafness. However, the underlying mechanism of Bap31 on X-linked syndrome remains unclear. Here, we found that a total of 21 proteins (9 up-regulated and 12 down-regulated proteins) related with X-linked syndrome were screened from shRNA-Bap31 transfected cells with the isobaric tags for relative and absolute quantification (iTRAQ) technique. One gene with the greatest change trend, monoamine oxidase A (MAOA), was identified. MAOA expression was up-regulated by Bap31 knockdown. However, Bap31 did not affect the ubiquitination degradation of MAOA protein. Of note, Bap31 selectively regulated the expression of cell division cycle associated 7-like (R1/RAM2/CDCA7L/JPO2, a transcriptional repressor of MAOA) and the binding activity of R1 with MAOA promoter, thereby affecting MAOA expression. This study demonstrates the molecular mechanisms of Bap31 in MAOA *via* R1 and supports the potential function of Bap31 on X-linked syndrome.

## Introduction

B-cell receptor-associated protein 31 (Bap31) gene is located from 153,700,492 to 153,724,746 bp on the long (q) arm of the X chromosome in humans (Adachi et al., 1996). The protein is a 28-kDa polytopic integral protein of the endoplasmic reticulum (ER), comprised of a hydrophobic N-terminus and an alpha-helical C-terminus contained with a KKXX motif (Adachi et al., 1996; Maatta et al., 2000). Bap31 is evolutionarily conserved and is known as Yet3p in yeast (Wilson and Barlowe, 2010). It transports certain membrane proteins from the ER to other cellular components, such as cellubrevin (Annaert et al., 1997), CD11b/CD18 (Zen et al., 2004), MHC-I (Abe et al., 2009) and protein tyrosine phosphatase-like B (Wang et al., 2004). A previous study showed that the caspase cleavage product of Bap31 (p20) induces the fission and apoptosis of mitochondria (Breckenridge et al., 2003). The study of conditional knockout mice of Bap31 indicates that Bap31 has functions in T-cell activation *via* T-cell receptor signaling (Niu et al., 2017). Bap31, as an important member of the ER-associated degradation (ERAD) (Wakana et al., 2008), promotes the mutant of cystic fibrosis transmembrane regulator (CFTRΔF508) to degradation *via* the Derlin1 complex (Wang et al., 2008). Our former study demonstrates that Bap31 regulates one of the central nervous system disease-related gene (valosin-containing protein, VCP) expression (Jia et al., 2018). Moreover, the contiguous deletion of ABCD1 DXS1357E (Bap31) leads to X-linked syndromes (Corzo et al., 2002; Iwasa et al., 2013). Mutations in Bap31 induce a severe X-linked phenotype, such as dystonia, deafness, and central hypomyelination (Cacciagli et al., 2013).

Monoamine oxidase A (MAOA) is an outer mitochondria membrane protein. Its mutation causes X-linked familial exudative vitreoretinopathy and Norrie disease (Chen et al., 1993, 1995). MAOA is a flavoenzyme that catalyzes the oxidative deamination of biogenic amines, such as serotonin, dopamine, and norepinephrine (Son et al., 2008). The substrates of MAOA are important factors in neural signal transmission; people with an abnormal expression of MAOA exhibit phenotypes, including autism (Verma et al., 2014), an aggressive behavior (Zhang et al., 2017) or depression (Gupta et al., 2016). The variable number of tandem repeats in the promoter region of MAOA frequently affects the expression of MAOA and induces the abnormal behaviors of males (Schluter et al., 2016; Manca et al., 2018). Moreover, cell division cycle associated 7-like (R1/RAM2/CDCA7L/JPO2) competitively binds the binding sites of *trans*-acting transcription factor 1 (SP1) with the human MAOA promoter, resulting in the down-regulation of the MAOA expression level (Ou et al., 2006a). Ou et al. (2006b) found that MAOA and its transcriptional repressor R1 participate in apoptotic signaling pathways. Furthermore, MAOA activates the Shh-IL6-Rankl signaling pathway in the promotion of prostate cancer metastasis (Wu et al., 2017).

The isobaric tags for relative and absolute quantification (iTRAQ) technique, one of the new proteomics approaches, has been widely used in the analysis of whole differential proteins among the different groups (Wiese et al., 2007). In the present study, iTRAQ was used to detect and analyze the whole differential proteins between shRNA-Bap31 transfected cells and control cells. In order to study the function of Bap31 on X-linked diseases, we screened the genes associated with X-linked diseases from the iTRAQ analyzed results. There were 21 proteins associated with X-linked diseases (9 up-regulated and 12 down-regulated proteins) regulated by Bap31. MAOA is one of them and up-regulated by Bap31 knockdown. Bap31 regulated the expression of MAOA *via* affecting the binding activity of R1 with the MAOA promoter. In conclusion, our results may demonstrate the mechanism of Bap31 on MAOA-associated X-linked diseases.

## Materials and Methods

### Cell Culture

N2a, HEK-293T, and SH-SY5Y cells were maintained in Dulbecco’s modified Eagle’s medium (DMEM, Gibco, MA, United States) with 10% fetal bovine serum (Hyclone, Life Technologies, CA, United States) and 1% pen-strep solution (Biological Industries, CT, United States) at 37°C in 5% CO_2_. The N2a cells that stably knock down Bap31 were cultured in DMEM medium with 10 μM puromycin (Thermo, MA, United States) under normal conditions. The SH-SY5Y cells stably expressing Bap31-Flag and the HEK293T cells stably expressing MAOA-HA were selected with 100 μg/ml G418/geneticin (Thermo) in DMEM medium under normal conditions.

### Plasmid Construction and Transfection

The fragments of R1 or MAOA-HA coding sequences were generated by polymerase chain reaction (PCR) and then ligated to pcDNA3.1(-) vector. The MAOA promoter fragment was amplified from HEK293T cells DNA and ligated to pGL3-basic plasmid. The shRNA fragments targeted to Bap31 or R1 were designed and subcloned into pLKO.1-puro plasmid. All plasmids were confirmed by sequencing before use (Genewiz Biotechnology Co., Ltd., Suzhou, China). Overexpression plasmids and/or shRNA vectors (2 μg) targeted to the specific genes were transfected by Lipofectamine 6000, provided by Beyotime Biotechnology (Shanghai, China), according to the manufacturer’s instructions. The cells transfected with control vectors were used as control groups.

### Dual Luciferase Reporter Assay

HEK-293T cells stably expressing MAOA-HA transfected with shRNA or overexpression plasmids (2 μg) targeted to Bap31 for 48 h. Then, the MAOA promoter fragment luciferase reporter plasmid (0.5 μg) and the phRL-SV40 vector (the transfection efficiency control, 0.05 μg) were co-transfected to the abovementioned cells with Lipofectamine 6000 for 48 h. The dual-luciferase assay kit (Beyotime) was used to detect the luciferase activities with a plate reader (BioTek, VT, United States). Three experiments were repeated and each group was set for three repeats. The firefly luciferase signal was normalized to the Renilla luciferase signal.

### Real-Time PCR

Total RNA was extracted from the different samples by using TRIzol reagent (Ambion, MA, United States). Two micrograms was synthesized to cDNA with the GoScript^™^ Reverse Transcription System (Promega, WI, United States) according to the manufacturer’s instructions. The mRNA levels of the genes were analyzed by the GoTaq^®^ qPCR Master Mix (Promega) with a CFX96 Touch^™^ Real-time PCR Detection System (Bio-Rad Laboratories, CA, United States). Three experiments were repeated and each group was set for three repeats. The results were analyzed by the 2^–ΔΔCt^ formula. The primers of the genes used in this study were shown in Table 1 (Genewiz Biotechnology Co., Ltd.).

**TABLE 1:** Primers used in this study.

Name	Forward primers (5′—3′)	Reverse primers (5′—3′)
Real-time PCR primers of MAOA (mouse)	cagtggagtggctacatgga	acatccttggactcaggctc
Real-time PCR primers of MAOA (human)	aattcagcggcttccaatgg	tttccgggcaagaatgaagc
Real-time PCR primers of Bap31 (mouse, human)	tccacatgaagcttttccgt	aaatgagagtcaccaggcgt
Real-time PCR primers of GAPDH (mouse)	agtgtttcctcgtcccgtag	gccgtgagtggagtcatact
Real-time PCR primers of GAPDH (human)	tcgtggaaggactcatgacc	atgatgttctggagagcccc
MAOA-HA plasmids primers	ggctagcatggagaatcaagagaaggc	ggaattctcaagcgtaatctggaacatcgtatgggtacatagaccgtggcaggagct
R1 plasmids primers	ggctagcatggagttggcgactcgct	cgggatccttaattgtcttctaccagctcc
shRNA-R1 plasmids primers	ccggcagacgacgtcaccgtatatcttcgaagatatacggtgacgtcgtctgtttttg	aattcaaaaacagacgacgtcaccgtatatcttcgaagatatacggtgacgtcgtctg
MAOA luciferase plasmids primers	ggctagcagctgccgacacaaggacatt	gaagatctcccttctatcaactcccccg
shRNA-Bap31 (human) plasmids primers	ccggatcgatgccgtgcgcgaaattct cgagaatttcgcgcacggcatcgattttttg	aattcaaaaaatcgatgccgtgcgcgaaattc tcgagaatttcgcgcacggcatcga
R1 element	agctgccgacacaaggacat	cccttctatcaactcccc

### Western Blot

Cells were lysed in RIPA buffer [1% TritonX-100, 50 mM Tris (pH 7.4), 150 mM NaCl, 1 mM EDTA, and 0.1% SDS] containing protease inhibitor cocktails (Thermo) and incubated on ice for 30 min. After centrifugation at 12,000 rpm for 15 min at 4°C, part of the supernatant was used to detect the protein concentration by an Enhanced BCA Protein Assay Kit (Beyotime) with a plate reader (BioTek, VT, United States) at 562 nm. 5 × SDS buffer was added to the remaining supernatant and then boiled for 10 min. Equal amounts of the total protein (30–50 μg) lysates were then separated by 8–10% SDS-PAGE and transferred onto 0.45 μM polyvinylidine fluoride membranes (Millipore, MA, United States). Then, the membranes were blocked with 5% skim milk for 1 h at room temperature, washed three times with Tris-buffered saline containing Tween-20 (TBST) buffer, incubated with primary antibodies at 4°C overnight, washed three times with TBST buffer, and incubated with the secondary antibodies for 1 h at room temperature. After that, the bands were visualized with a ChemiDoc^™^ XRS + system (Bio-Rad Laboratories, CA, United States) using an ECL detection kit (Tanon, Shanghai, China). The intensities of the bands targeted to the proteins were calculated and normalized to that of β-actin using Image Lab software (Bio-Rad). Three experiments were repeated. The antibodies used in this study were as follows: rabbit anti-MAOA (Abcam, MA, United States), rabbit anti-R1 (Abcam), rabbit anti-Sp1 (Wanlei Biotechnology Co., Ltd., Shenyang, China), mouse anti-ub (Santa Cruz Biotechnology, TX, United States), mouse anti-β-actin (Abcam), chicken anti-Bap31, mouse anti-HA (Abcam), and the secondary antibodies (Thermo).

### Immunoprecipitation

HEK-293T cells overexpressing MAOA-HA were transfected with shRNA or overexpression plasmids (2 μg) targeted to Bap31 for 48 h. The whole-cell lysates were lysed in an immunoprecipitation lysis buffer (Beyotime Biotechnology) containing a protease inhibitor and centrifuged at 4°C for 15 min at 12,000 rpm. The supernatant was incubated with mouse anti-HA (Abcam) antibody overnight at 4°C and then incubated with the protein A/G Sepharose (Beyotime) for 4°C overnight. The immunoprecipitated proteins were washed three times with lysed buffer, and equal amounts of total protein (50 μg) lysates were eluted with 1 × SDS loading buffer and boiled for 10 min. Western blot was used to analyze the samples.

### Flow Cytometry

The cells were resuspended in 200 μl of 2% paraformaldehyde (PFA) in phosphate buffer saline (PBS), incubated on ice for 15 min, and then stained with anti-MAOA diluted in PBS with 1% saponin on ice water bath at 4°C for 1 h, followed by the fluorescein-isothiocyanate (FITC)-conjugated secondary antibody on ice water bath at 4°C for 45 min. The solutions were washed three times with PBS containing 2% bovine serum albumin (BSA), resuspended in PBS, and then detected with a flow cytometry (Becton Dickinson, NJ, United States).

### Protein Digestion, TMT Labeling, and LC-MS/MS Analysis

All samples were homogenized in a lysis buffer [8 M urea, 1% Triton-100, 10 mM dithiothreitol (DTT), and 1% protease inhibitor]. After centrifugation at 20,000 × *g* for 10 min at 4°C, the supernatant was precipitated with cold 15% TCA for 2 h at −20°C, followed by centrifugation at 4°C for 10 min. The precipitate was washed three times with cold acetone and then re-dissolved in buffer [8 M urea and 100 mM triethylammonium bicarbonate (TEAB), pH 8.0], and the protein concentration was determined with the BCA protein assay reagent (Beyotime).

For digestion, the protein solution was reduced with 10 mM DTT (Sigma, NY, United States) at 37°C for 1 h and alkylated with 20 mM iodoacetamide (Sigma) in darkness at room temperature for 45 min. Then, the protein sample was diluted by adding 100 mM TEAB (Applied Biosystems, Milan, Italy) to a urea (Sigma) concentration of less than 2 M. Finally, trypsin (Promega) was added at a ratio of 1:50 to the proteins for the first digestion overnight, followed by a second 4-hour digestion with a 1:100 trypsin-to-protein mass ratio. Then, 100 μg protein for each sample was digested with trypsin for the following experiments. The peptide was desalted by Strata X C18 SPE column (Phenomenex, CA, United States), vacuum-dried, and reconstituted in 0.5 M TEAB and labeled according to the manufacturer’s protocol for 6-plex TMT kit (Thermo). The protein samples were labeled as 130 (control-1), 129 (control-2), 131 (control-3), 131 (shRNA-Bap31-1), 128 (shRNA-Bap31-2), and 130 (shRNA-Bap31-3).

The sample was then fractionated into 80 fractions by high-pH reverse-phase HPLC using Agilent 300Extend C18 4.6 × 100 mm column (5 μm, Agilent, CA, United States) with a gradient of 2 to 60% acetonitrile in 10 mM ammonium bicarbonate, pH 10, over 80 min. Then, the peptides were combined into 18 fractions and dried by vacuum centrifuging.

The peptides were dissolved in 0.1% FA and loaded onto a reversed-phase pre-column (Acclaim PepMap 100, Thermo Scientific). Peptide separation was performed using a reversed-phase analytical column (Acclaim PepMap RSLC, Thermo Scientific) at a constant flow rate of 280 nl/min on an EASY-nLC 1000 ultra-performance liquid chromatography (UPLC) system. The gradient was comprised of an increase from 7 to 20% solvent B (0.1% FA in 98% ACN) over 24 min, 20 to 35% in 8 min, 35 to 80% in 5 min, and holding at 80% for 3 min. The resulting peptides were analyzed with a Q Exactive^™^ hybrid quadrupole-Orbitrap mass spectrometer (ThermoFisher Scientific).

The peptides were analyzed by tandem mass spectrometry (MS/MS) in Q Exactive^™^ (Thermo) coupled to the UPLC. The intact peptides were detected in the Orbitrap at a resolution of 70,000. The peptides were selected for MS/MS using NCE settings as 27, 30, and 33. The resolution of ion fragments detected in the Orbitrap was 17,500. A data-dependent procedure that alternated between one MS scan followed by 20 MS/MS scans was applied for the top 20 precursor ions above a threshold ion count of 1.0E4 in the MS survey scan with 30.0 s dynamic exclusion. The electrospray voltage applied was 2.0 kV. Automatic gain control was used to prevent overfilling of the ion trap, and 5E4 ions were accumulated for generation of MS/MS spectra. For MS scans, the m/z scan range was 350 to 1,800. The fixed first mass was set as 100 m/z.

### Chromatin Immunoprecipitation

HEK-293T cells were co-transfected with shRNA-Bap31 and R1 over-expression plasmids or Bap31 overexpression plasmids and shRNA-R1 plasmids for 48 h. ChIP assay was performed according to the manufacturer’s protocol (Beyotime). The ChIP primer (Table 1) sets were checked for linear amplification and designed to amplify the region of the human MAOA promoter.

### Database Search and Bioinformatics Analysis

Proteins with a fold change of 1.5 (*p* < 0.05) between the two groups were identified as differentially expressed. The resulting MS/MS data were processed using Mascot search engine (v.2.3.0). The results of the tandem mass spectra were searched against the Uniprot mouse database (16,717 sequences). The differentially regulated proteins were classified by Gene Ontology (GO) annotation derived from the UniProt-GOA database into three categories: biological process, cellular compartment, and molecular function. The subcellular and gene localization of differentially regulated proteins was analyzed.

### Immunofluorescence

For the immunofluorescence stain, the cells were fixed with 2% PFA in PBS buffer for 30 min, washed with PBS three times, and permeated with 0.2% Triton X-100 for 30 min at room temperature. After that, the cells were washed with PBS three times, blocked with 2% BSA in PBS buffer and then stained with an anti-R1 antibody followed by the FITC-conjugated secondary antibody (Abcam). The nuclei were stained with Hoechst 33342 (Sigma-Aldrich). The fluorescence images were taken under a confocal laser scanning microscope (Leica, IL, United States). The fluorescence intensity was analyzed with the ImageJ software (NIH).

### Statistical Analysis

Independent-sample Student’s tests and one-way ANOVA were used to analyze the significance of the results. All experimental data were reported as the mean ± standard error of the mean (SEM) (significance: ^∗^*p* < 0.05, ^∗∗^*p* < 0.01).

## Results

### The Proteins Involved in X-Linked Diseases Regulated by Bap31

**FIGURE 1:**
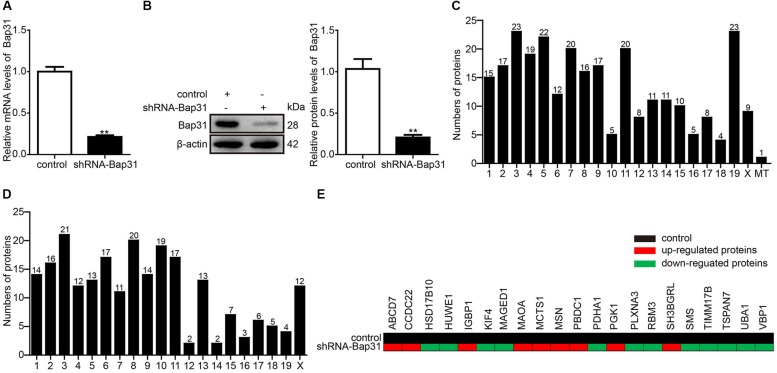
The located chromosome and X-linked Bap31 regulated proteins. Real-time PCR **(A)** and Western blot **(B)** analyses were used to detect the expression levels of Bap31 in shRNA-Bap31 transfected N2a cells. The isobaric tags for relative and absolute quantification technique was used to detect and analyze the different proteins between shRNA-Bap31 transfected cells and control cells. The numbers of up-regulated **(C)** and down-regulated **(D)** proteins were analyzed according to the differently located chromosomes in shRNA-Bap31 transfected cells. The genes regulated by Bap31 knockdown were located on the X chromosome **(E)**. Black: control, red: up-regulated genes, green: down-regulated genes. **p < 0.01 vs. control groups, *n* = 3.

Bap31 is widely expressed in various cells, such as B cells (Adachi et al., 1996), T cells (Niu et al., 2017), and neurons (Manley and Lennon, 2001). To explore the unknown function of Bap31, we used the Bap31 knockdown plasmid (pL/shRNA-mouse-shRNA-Bap31) and the antibiotics to construct Bap31 knockdown cells (shRNA-Bap31). Real-time PCR and Western blot were used to analyze the knockdown efficiency of Bap31; the mRNA and protein levels of Bap31 were significantly decreased by 79 and 80% compared with that of the control cells, respectively (Figures 1A,B). These results demonstrate that the shRNA-Bap31 transfected cells are qualified as Bap31 effectively knockdown cells.

Isobaric tags for relative and absolute quantification is a recently developed technique in the quantitative proteomics which measures fold changes in protein expression among different groups (Moulder et al., 2018). In this study, iTRAQ was used to detect the differentially expressed proteins between shRNA-Bap31 transfected cells and control cells. In total, 4,349 proteins were identified and 2,989 proteins were quantified. When setting the quantification ratio (shRNA-Bap31 transfected cells/control cells) of >1.5 as up-regulated threshold and <0.67 as down-regulated threshold (*p* < 0.05), there were 276 up-regulated and 228 down-regulated proteins (Table 2). Then, we analyzed and counted the subcellular location, biological process, cellular component, and molecular function of Bap31-regulated proteins (Supplementary Tables S1–S4). The results indicate that Bap31 affected many important biological processes.

**TABLE 2:** Summary of identified, quantified proteins and differentially quantified proteins (>1.5, or <0.67).

Name	Identified	Quantified	Up-regulated (>1.5)	Down-regulated (<0.67)
Protein numbers	4,349	2,989	276	228

It is well recognized that Bap31 gene is located on the X chromosome, and its mutation causes X-linked diseases (Osaka et al., 2012; Cacciagli et al., 2013). To explore the mechanism of Bap31 on X-linked diseases, we identified 21 X-linked diseases-related proteins contained with 9 up-regulated (ABCB7, CCDC22, IGBP1, MAOA, MCTS1, MSN, PBDC1, PGK1, and SH3BGRL) and 12 down-regulated (HSD17B10, HUWE1, KIF4, MAGED1, PDHA1, PLXNA3, RBM3, SMS, TIMM17B, TSPAN7, UBA1, and VAP1) proteins from the iTRAQ results (Figures 1C–E and Supplementary Table S5). The findings support the interaction between Bap31 and X-linked diseases.

### Bap31 Negatively Regulates the Expression of MAOA

**FIGURE 2:**
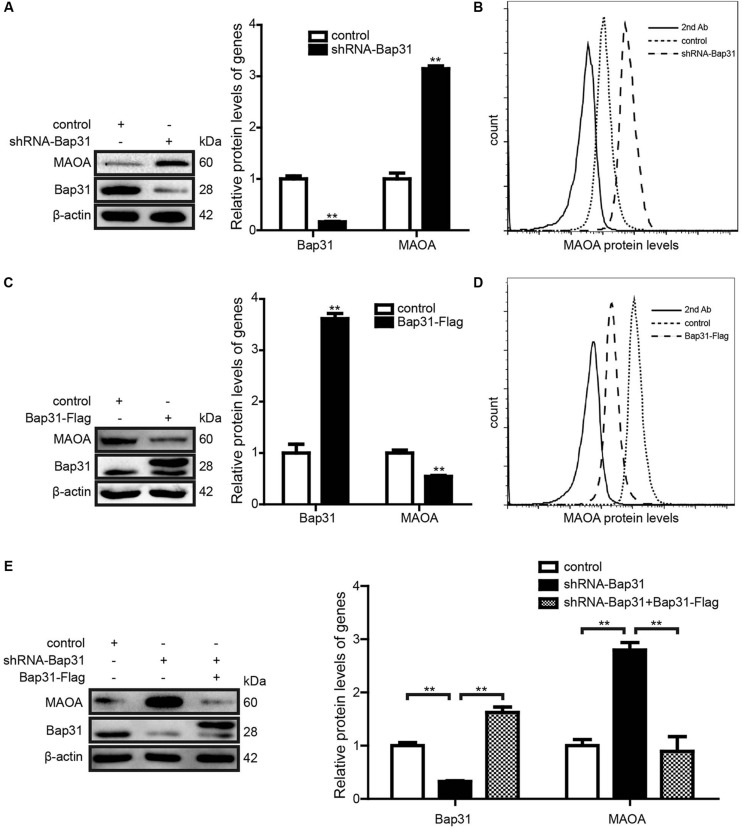
The expression levels of monoamine oxidase A (MAOA) in shRNA-Bap31 and Bap31-Flag transfected cells. Western blot analyses were used to detect the protein levels of MAOA in shRNA-Bap31 **(A)** and Bap31-Flag **(C)** transfected cells. **(B,D)** Flow cytometry was used to detect the levels of MAOA in the same group as in panels **(A,C)**. The protein levels of MAOA in shRNA-Bap31 transfected cells with overexpression of Bap31-Flag **(E)**. The histogram shows the relative expression levels of targeted proteins in Bap31 disturbed groups compared with the control groups. **p < 0.01 vs. control groups, *n* = 3.

Monoamine oxidase A is one of the Bap31-regulated proteins and up-regulated by the knockdown of Bap31 (Figure 1E). To further verify the results, Western blot analysis was used to detect the expression level of MAOA in shRNA-Bap31 transfected cells. The results showed that the protein level of MAOA was 3.14-fold in shRNA-Bap31 transfected cells compared with that of the control cells (Figure 2A). Meanwhile, we also constructed Bap31 overexpression cells transfected with Bap31-Flag plasmid and detected the expression level of MAOA. The results showed that the protein level of MAOA was 54.5% in Bap31 overexpression cells compared with that in control cells (Figure 2C). Besides that, we overexpressed Bap31-Flag in shRNA-Bap31 transfected cells, and Western blot analysis showed that the overexpressed Bap31 reversed the MAOA expression level which was increased by Bap31 knockdown (Figure 2E). Flow cytometry was used to further confirm that the levels of MAOA in Bap31 disturbed cells were significantly changed compared with that in the control cells; the same change trend as that of Western blot analysis was observed (Figures 2B,D). These results indicate that Bap31 negatively regulates the expression of MAOA.

### Bap31 Does Not Affect the Ubiquitination and Degradation Ratio of MAOA

**FIGURE 3:**
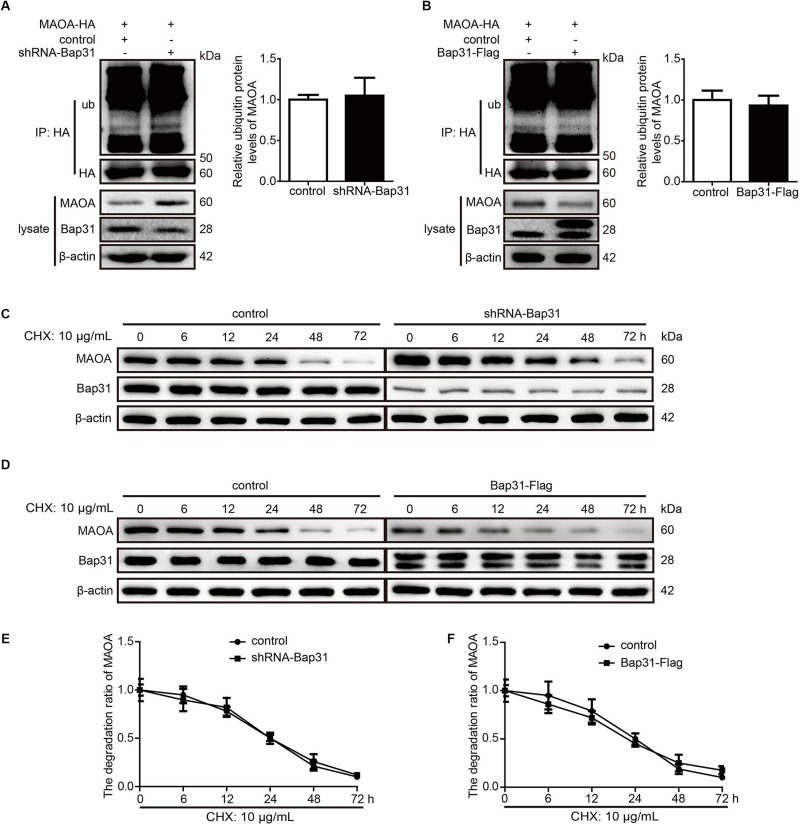
Bap31 did not regulate the degradation of monoamine oxidase A (MAOA). MAOA-HA stable expression cells were transfected with shRNA-Bap31 **(A)** or Bap31-Flag **(B)** plasmids for 48 h. The immunoprecipitated proteins were detected and showed the ubiquitinated MAOA levels. The histogram indicates the relative expression levels of ubiquitinated MAOA in Bap31 disturbed groups compared with the control groups. The same cells as in panels **(A,B)** were treated with 10 μg/ml CHX and then the protein levels of MAOA were determined at 0, 6, 12, 24, 48, and 72 h **(C,D)**. The line chart indicated the degradation ratio of MAOA in Bap31 disturbed groups and control groups cultured with CHX **(E,F)**, *n* = 3.

Monoamine oxidase A can be degraded by Rines E3 ubiquitin ligase (Kabayama et al., 2013), leading us to speculate that one possibility for the mechanism may be that Bap31 affected the ubiquitin-dependent proteasomal degradation of MAOA. To further explore this mechanism, we constructed the HEK293T cell line stably transfected with MAOA-HA plasmid (Supplementary Figure S1). After that, we transfected shRNA-Bap31 to the cells and used an anti-HA antibody to pull down MAOA to exclude the effect of Bap31 on the regulation of endogenous MAOA mRNA levels. Western blot analysis of precipitated proteins with an anti-ub antibody showed that the ubiquitinated MAOA in shRNA-Bap31 transfected cells was not different from that in control cells (Figure 3A); the same results were obtained in Bap31 overexpressed cells (Figure 3B). The results demonstrated that Bap31 did not directly affect the ubiquitination of MAOA. Moreover, we detected the protein levels of MAOA and analyzed the degradation ratio in shRNA-Bap31 transfected cells cultured with 10 μg/ml cycloheximide (CHX, one of the protein synthesis inhibitors) for 0, 6, 12, 24, 48, and 72 h. The results showed that the degradation ratio of MAOA was not significantly changed by the knockdown of Bap31 (Figures 3C,E). Meanwhile, we detected the levels of MAOA in the Bap31 overexpressed cells. The results showed that the degradation ratios of MAOA either in the presence of Bap31-Flag or in the empty vector transfected cells were not significantly different (Figures 3D,F). Together, these results indicate that Bap31 does not directly affect the degradation of MAOA protein.

### Bap31 Regulates the mRNA Levels of MAOA via R1

After ruling out a direct effect of Bap31 on the degradation of MAOA, we compared the amount of mRNA levels of MAOA in shRNA-Bap31 transfected cells and control cells. Real-time PCR analyses showed that the mRNA level of MAOA in shRNA-Bap31 transfected cells was 2.53-fold compared with that of the control cells (Supplementary Figure S2).

**FIGURE 4:**
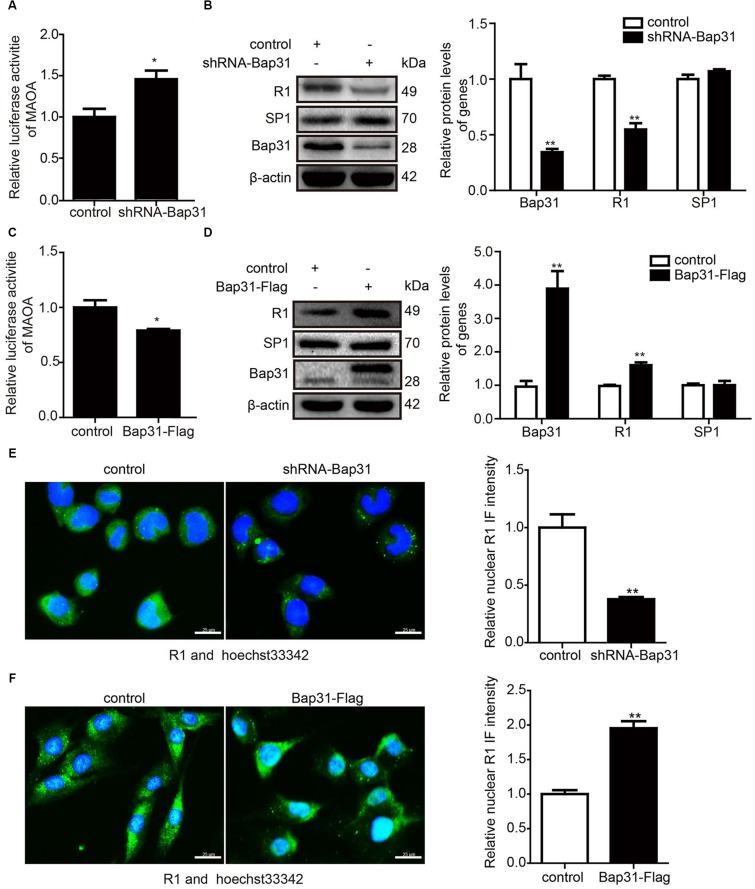
Bap31 regulated the expression levels of R1. The luciferase activities of the monoamine oxidase A promoter were detected in shRNA-Bap31 **(A)** or Bap31-Flag **(C)** transfected cells. Western blot analyses were used to detect the protein levels of SP1 and R1 in shRNA-Bap31 **(B)** or Bap31-Flag **(D)** transfected cells. The histogram shows the relative expression levels of the targeted protein in Bap31 disturbed groups compared with the control groups. Immunofluorescent staining was further used to confirm the levels of R1 in the same groups as in panels **(B,D)**. The merged images (green: R1, blue: hoechst stain for nucleus) are shown. The histogram shows the relative expression levels of the nuclear R1 protein in Bap31 disturbed groups compared with the control groups **(E,F)**. *p < 0.05, **p < 0.01 vs. control groups, *n* = 3.

SP1 (the transcription factor of MAOA) and R1 (the transcriptional repressor of MAOA) competitively bind with the MAOA promoter region and reversely regulate the expression of MAOA (Ou et al., 2006a). To further explore the mechanism of Bap31 on the mRNA level of MAOA, we constructed the MAOA luciferase report plasmid containing the binding sites of SP1 and R1. Then, we transfected it into Bap31 disturbed cells. The results showed that the luciferase activities of MAOA were negatively regulated by Bap31 (Figures 4A,C). Western blot analyses showed that the protein level of R1 in shRNA-Bap31 transfected cells was 54.8% of that in the control cells (Figure 4B), and the protein level of R1 in Bap31-Flag overexpressed cells was 1.60-fold of that in the control cells (Figure 4D). Immunofluorescent staining analysis obtained the same change trend as that of Western blot analysis (Figures 4E,F). However, the protein levels of SP1 did not have a significant difference among these groups (Figures 4B,D). These results imply that Bap31 regulates MAOA possibly *via* the regulation of R1.

**FIGURE 5:**
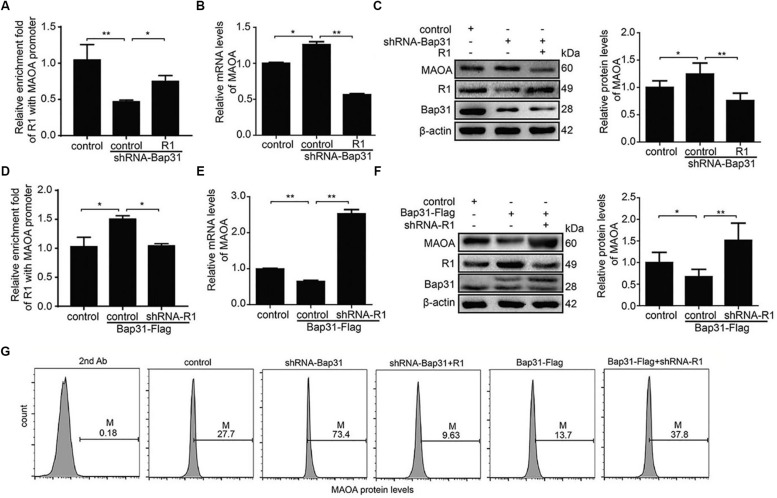
Bap31 regulated monoamine oxidase A (MAOA) expression through R1. ChIP was used to analyze the binding activities between R1 and MAOA promoter in Bap31 disturbed cells with R1 reverse interference **(A,D)**. Real-time PCR and Western blot were used to detect the mRNA and the protein levels of MAOA in shRNA-Bap31 transfected cells with over-expressing R1 **(B,C)** or in Bap31-Flag transfected cells with knockdown of R1 **(E,F)**. **(G)** Flow cytometry was used to detect the levels of MAOA in the same groups as in panels **(A,D)**. The histogram shows the relative expression levels of the targeted protein. *p < 0.05, **p < 0.01 vs. control groups, *n* = 3.

To further explore the mechanism of Bap31 on MAOA *via* R1, we constructed R1 overexpression and shRNA-R1 plasmids and transfected them into Bap31 disturbed cells. Then, we used ChIP assay to explore whether Bap31 could impact MAOA gene transcription *via* R1. Our data revealed that the overexpression of R1 increased the binding activities of R1 to the MAOA promoter region which was reduced by the knockdown of Bap31 (Figure 5A). Conversely, the binding activities of R1 to the MAOA promoter region was increased by the overexpression of Bap31, and the knockdown of R1 in Bap31 over-expressed cells reduced the activities compared with the non-knockdown R1 groups (Figure 5D). Moreover, real-time PCR and Western blot were used to detect the levels of MAOA in Bap31 knockdown cells with overexpression of R1; the results showed that they were 44.7 and 60.9% compared with that of Bap31 knockdown cells, respectively (Figures 5B,C). Conversely, we inhibited R1 by transfection shRNA-R1 plasmid into Bap31 over-expressed cells. The mRNA and protein levels of MAOA in these cells were 3.93- and 2.26-fold compared to the Bap31 over-expressed cells (Figures 5E,F). Furthermore, the results of flow cytometry showed the same change trend as that of real-time PCR and Western blot analyses (Figure 5G). These results demonstrate that Bap31 regulates the levels of MAOA *via* R1.

## Discussion and Conclusion

Bap31 is highly expressed in the ER and its gene is located on the X chromosome (Adachi et al., 1996). It participates in transporting member proteins from the ER to other organelles, mediating apoptosis *via* p20 and regulating the ERAD pathway (Breckenridge et al., 2003; Wakana et al., 2008). Our recent study indicates that Bap31 deficiency leads to the formation of amyloid-β plaques *via* reducing RTN3 stability in Alzheimer’s disease (Wang et al., 2019). The conditional knockout of BAP31 in hepatocyte promotes SREBP1C activation and hepatic lipid accumulation and worsens insulin resistance in HFD-induced obesity in mice (Xu et al., 2018). Bap31 regulates the expression of VCP *via* its transcription factor Elf2 (Jia et al., 2018). However, there is still little research on the biological function of Bap31. ITRAQ-based proteomic technique as a new technique is used to detect the whole proteomics among different groups. In this study, iTRAQ was used to detect and analyze the proteins regulated by Bap31. In analyzing the results, there are 504 differentially expressed proteins in Bap31 knockdown cells: 276 up-regulated and 228 down-regulated proteins. These proteins participated in many important biological process and cellular component and molecular function, for example, biological regulation, metabolic process, cellular component organization or biogenesis, multicellular organismal process, developmental process, immune system process, and so on (Supplementary Tables S1-S5). The results of iTRAQ provided the basis for studying the unknown function of Bap31.

The contiguous deletion of SLC6A8 and Bap31 is related to hearing loss and liver dysfunction (Osaka et al., 2012). Bap31 mutations cause a severe X-linked phenotype with deafness, dystonia, and central hypomyelination (Cacciagli et al., 2013). Besides that, the contiguous ABCD1 DXS1357E (Bap31) deletion syndrome (CADDS) is 90 kb, ranging from exon 4 of Bap31 to exon 8 of PDZD4 and contained ABCD1, PLXNB3, SRPK3, IDH3G, and SSR4 (Corzo et al., 2002). Based on these, we speculated that Bap31 may be an important factor in mediating X-linked diseases. In this study, the analysis of the 504 differentially expressed proteins regulated by Bap31 showed that there were 21 genes located on the X chromosome containing 9 up-regulated and 12 down-regulated proteins (Figure 1). Our results further demonstrated the function of Bap31 on X-linked diseases.

Monoamine oxidase A is a flavoenzyme located on the outer mitochondrial membrane. It can catalyze the oxidative deamination of serotonin, dopamine, and norepinephrine (Yu et al., 2014). Mutations of MAOA cause X-linked diseases (Chen et al., 1993) and increased risk for Parkinson’s disease (Hotamisligil et al., 1994) and Norrie disease (Wolff et al., 1992). Moreover, MAOA participates in apoptotic signaling pathways (Ou et al., 2006b) and Shh-IL6-Rankl signaling pathway (Wu et al., 2017). However, whether Bap31 regulates the expression of MAOA was unknown. Our results showed that Bap31 negatively regulates the expression of MAOA, whether knockdown or overexpression of Bap31 (Figure 2). To explore the mechanism of Bap31 on MAOA, we firstly detected the regulation of Bap31 on the degradation of MAOA. Although Bap31 affects the degradation of many proteins (Wang et al., 2008; Chen et al., 2019), the regulation of Bap31 on MAOA expressions does not appear to be dependent on these effects. Bap31 did not directly affect the ubiqutin-mediated degradation of MAOA in MAOA-HA stable cells with knockdown or overexpression of Bap31 (Figures 3A,B). Consistent with this, Bap31 hardly changed the degradation ratio of MAOA compared to its controls (Figures 3C–F). It suggests that Bap31 does not exert its direct effect on the MAOA protein degradation level. Rather intriguingly, the depletion of Bap31 with shRNA-Bap31 strongly increased the amounts of mRNA for MAOA (Supplementary Figure S2). Furthermore, Bap31 did not affect the ubiquitin-mediated degradation level of transfected MAOA-HA protein expression driven by a CMV promoter in the pcDNA plasmid (Figures 3A,B). These led us to further verify that Bap31 mediates the transcription of MAOA. R1, the transcriptional repressor of MAOA, was down-regulated and caused apoptosis in serum starvation (Ou et al., 2006b). Consistent with the former study, our data showed that knockdown or overexpression of R1 affected the binding activities of R1 with the MAOA promoter, thereby negatively regulating the mRNA and protein levels of MAOA. Besides that, our results also indicated that Bap31 regulates the expression levels of R1 (Figure 4). Ou et al. (2006b) have found that Bcl-2 was decreased and caused the down-regulation of the level of R1 in serum starvation-induced apoptosis. Previous studies indicate that Bap31 is identified as a Bcl-2 interacting protein (Ng et al., 1997) and the cleavage of Bap31 (p20) as the upstream of Bcl-2 affects cell surface properties (Stojanovic et al., 2005). One of the possible mechanisms of Bap31 on R1 may be *via* Bcl-2. However, the molecular mechanism of Bap31 on R1 still needs further exploration.

In summary, our findings indicate that Bap31 regulates MAOA possibly *via* its effect on R1 and support the functions of Bap31 in X-linked diseases. Our work helps to uncover the molecular mechanism of Bap31 on X-linked diseases and may help find the potential molecular targets for X-linked disease therapy.

## Data Availability Statement

The raw data supporting the conclusions of this manuscript will be made available by the authors, without undue reservation, to any qualified researcher.

## Author Contributions

CJ wrote the manuscript and researched the data. GL researched and analyzed the data. RJ helped with revising the discussion. XL and QY helped with analyzing the data. WL helped with revising the manuscript. YH directed the study and revised the manuscript. BW directed the study, analyzed and approved all of the data, and edited the manuscript.

## Conflict of Interest

The authors declare that the research was conducted in the absence of any commercial or financial relationships that could be construed as a potential conflict of interest.
