# Supplementary material for: B-Cell Receptor-Associated Protein 31 Negatively Regulates the Expression of Monoamine Oxidase A Via R1
Source: Front Mol Biosci. 2020 Apr 30;7:64. doi: 10.3389/fmolb.2020.00064 (PMC7212379; doi:10.3389/fmolb.2020.00064)
Supplement: Supplementary file 1 [file Data_Sheet_1.docx]

Supplementary Material

**B****-cell Receptor-Associated Protein 31 Negatively Regulates the Expression of Monoamine Oxidase A *Via* R1**

Cong-cong Jia^1, 2, 3†^, Guoxun Li^1†^, Rui Jiang^1^, Xia Liu^1^, Qing Yuan^1^, Weidong Le^2, 3^, Yue Hou^1, 4^^*^, Bing Wang^1*^

*^1^College of Life and Health Sciences, Northeastern University, Shenyang, China.*

*^2^Center for Clinical Research on Neurological Diseases, The First Affiliated Hospital, Dalian Medical University, Dalian, China.*

*^3^Liaoning Provincial Key Laboratory for Research on the Pathogenic Mechanisms of Neurological Diseases, The First Affiliated Hospital, Dalian Medical University, Dalian, China.*

*^4^Key Laboratory of Data Analytics and Optimization for Smart Industry, Ministry of Education, Northeastern University, Shenyang, China.*

*** *Correspondence:*** *wangbing@mail.neu.edu.cn;* [*houyue@mail.neu.edu.cn*](mailto:houyue@mail.neu.edu.cn)

**^†^ *Contributed equally***

# Supplementary Figures


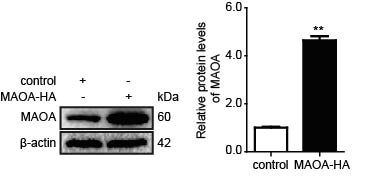


**FIGURE S1** **The** **protein** **levels** **of** **MAOA** **in** **MAOA-HA** **transfected cells.**

HEK-293T cells were transfected with control vector or MAOA-HA plasmids, the protein levels of MAOA were detected by Western blot in these groups. Histograms showed the relative change of MAOA, ***p*<0.01 vs. control groups, n = 3.


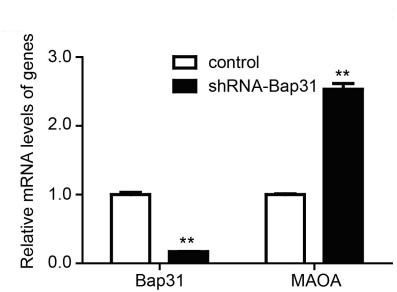


**FIGURE** **S2** **The** **mRNA** **levels** **of** **MAOA** **in** **shRNA-Bap31** **transfected** **cells.**

Real-time PCR was used to detect the mRNA levels of MAOA in shRNA-Bap31 transfected cells. ***p*<0.01 vs. control groups, n = 3.

**Supplementary Table S1 Distribution statistics of differential proteins in biological processes**

| **Biological process** | **Numbers of up-regulated proteins** | **Numbers of down-regulated proteins** |
| --- | --- | --- |
| cellular process | 212 | 182 |
| single-organism process | 181 | 130 |
| biological regulation | 140 | 100 |
| metabolic process | 131 | 143 |
| response to stimulus | 107 | 66 |
| localization | 94 | 45 |
| cellular component organization or biogenesis | 82 | 77 |
| multicellular organismal process | 79 | 54 |
| developmental process | 67 | 50 |
| signaling | 52 | 24 |
| immune system process | 31 | 0 |
| other | 59 | 60 |

**Supplementary Table S2 Distribution statistics of differential proteins in cellular component**

| **Cellular component** | **Numbers of up-regulated proteins** | **Numbers of down-regulated proteins** |
| --- | --- | --- |
| cell | 247 | 209 |
| organelle | 192 | 189 |
| membrane | 130 | 76 |
| macromolecular complex | 75 | 89 |
| membrane-enclosed lumen | 34 | 51 |
| extracellular region | 25 | 0 |
| cell junction | 18 | 0 |
| other | 13 | 34 |

**Supplementary Table S3 Distribution statistics of differential proteins in molecular function**

| **Molecular function** | **Numbers of up-regulated proteins** | **Numbers of down-regulated proteins** |
| --- | --- | --- |
| binding | 197 | 168 |
| catalytic activity | 129 | 93 |
| enzyme regulator activity | 27 | 7 |
| transporter activity | 17 | 15 |
| molecular transducer activity | 9 | 0 |
| structural molecule activity | 0 | 15 |
| other | 28 | 22 |

**Supplementary Table S4 The statistics of differential protein in subcellular organism**

| **Subcellular organism** | **Numbers of up-regulated proteins** | **Numbers of down-regulated proteins** |
| --- | --- | --- |
| cytosol | 128 | 68 |
| nuclei | 54 | 83 |
| mitochondria | 26 | 25 |
| plasma membrane | 17 | 18 |
| cytosol-nuclear | 13 | 8 |
| cytoskeleton | 3 | 4 |
| endoplasmic reticulum | 4 | 7 |
| extracellular | 27 | 16 |
| peroxisome | 2 | 0 |
| cytosol-mitochondria | 1 | 0 |
| lysosome | 1 | 0 |

**
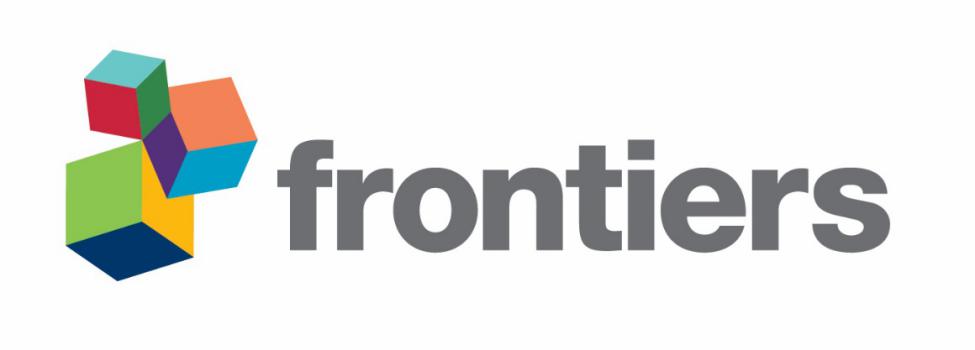
**
